# Prevalence of Biofilm-Forming and Antibiotic-Resistant Coagulase-Negative Staphylococci Isolated from Hospitalized Patients in an Orthopedic Clinic

**DOI:** 10.3390/pathogens15010120

**Published:** 2026-01-21

**Authors:** Tatiana Szabóová, Gabriela Gregová, Ján Király, Nikola Dančová, Vanda Hajdučková, Patrícia Hudecová, Simona Hisirová, Peter Polan, Viera Lovayová

**Affiliations:** 1 Department of Public Veterinary Medicine and Animal Welfare, The University of Veterinary Medicine and Pharmacy in Košice, 041 81 Košice, Slovakia; tatiana.szaboova@uvlf.sk (T.S.); gabriela.gregova@uvlf.sk (G.G.);; 2 Department of Microbiology and Immunology, The University of Veterinary Medicine and Pharmacy in Košice, 041 81 Košice, Slovakiasimona.hisirova@student.uvlf.sk (S.H.); 3 Faculty Hospital AGEL Košice-Šaca a.s., Lúčna 57, 040 15 Košice, Slovakia; 4 Department of Medical and Clinical Microbiology, Faculty of Medicine, Pavol Jozef Šafárik University in Košice, Trieda SNP 1, 040 11 Košice, Slovakia

**Keywords:** staphylococci, CoNS, biofilm formation, antimicrobial resistance, efflux pump

## Abstract

Methicillin-resistant coagulase-negative staphylococci (MRCoNS) are a major cause of infectious diseases, owing to their ability to form biofilms and colonize community and hospital environments. MRCoNS strains were identified using biochemical tests, an MALDI-TOF MS analyzer, and PCR-based 16S rRNA gene confirmation. This study was designed to assess antibiotic resistance and biofilm-forming capacity and to determine the presence of the *mecA*, *mecC*, *agrA*, *srtA*, *icaABCD*, *bap*, *fnbAB*, and *clfAB* genes in MRCoNS isolates. From patients undergoing random screening during hospitalization in the Orthopedics Clinic in Slovakia, 28 strains of MRCoNS were identified: *S. epidermidis* (*n* = 10), *S. hominis* (*n* = 8), *S. haemolyticus* (*n* = 4), *S. lugdunensis* (*n* = 3), while *S. simulans*, *S. pasteuri*, and *S. warneri* were detected only once. The highest rates of resistance were observed for ampicillin, oxacillin, rifampicin, trimethoprim (100%), and erythromycin (62%). The *mecA* gene was detected in 12 analyzed isolates. In 12 isolates, MDR, strong efflux pump activity, and strong or moderate biofilm formation were simultaneously detected. Our findings highlight the problems posed by biofilm-forming, resistant CoNS in hospitalized patients and the importance of diagnostics, separation, rapid treatment, and proper hospital hygiene.

## 1. Introduction

CoNS are the most abundant nonpathogenic commensals of the normal human skin microbiota and mucous membranes [1]. They represent a diverse group of commensal yet opportunistic pathogens that colonize humans with reported prevalence rates ranging from approximately 30% to nearly 100% [2,3]. In orthopedic settings, CoNS have paradoxically emerged as leading etiologic agents of hospital-acquired and implant-associated infections, including prosthetic joint infections and postoperative surgical site infections, due to their ability to adhere to biomaterials and form biofilms [4]. Musculoskeletal infections include osteomyelitis, infectious arthritis, spondylodiscitis, fracture-related infections, and periprosthetic joint infections [5]. The use of implants further increases the risk of infection, with *Staphylococcus aureus* and CoNS being the most frequent pathogens [6]. The predominant CoNS species in orthopedic infections include *S. epidermidis*, *S. hominis*, *S. pasteuri*, *S. warneri*, *S. lugdunensis*, *S. haemolyticus*, and *S. schleiferi* [7]. The ability of these organisms to persist on implant surfaces complicates treatment and contributes to recurrent or chronic infections [8,9]. *S. epidermidis*, *S. haemolyticus*, and *S. hominis* are the CoNS most frequently implicated in bloodstream infections, prosthetic device-related infections, and postoperative complications [10,11]. Infections associated with orthopedic implants may cause serious surgical complications. Approximately 5% of elective and emergency orthopedic surgeries result in postoperative infections involving orthopedic implants. *S. epidermidis* and *S. aureus* are the most common causes of implanted medical device infections involving robust biofilm formation. Together, they account for up to 50% of heart valve infections and up to 70% of catheter-associated biofilm infections in hospitalized patients [12,13].

The rising prevalence of antimicrobial resistance has greatly impacted hospital-acquired infections caused by CoNS. Oxacillin is routinely used for susceptibility testing and treatment, with clinical studies reporting resistance rates of 66–95% among CoNS isolates [14,15]. Resistance to β-lactams, macrolides, aminoglycosides, fluoroquinolones, and even glycopeptides represents a major global concern [16]. Antibiotic resistance in CoNS can arise via genetic mutations, horizontal gene transfer, enzymatic inactivation, target modifications, efflux pumps, and biofilm formation [17,18]. These mechanisms confer resistance to beta-lactams, aminoglycosides, macrolides, and newer agents, often mediated by mobile elements such as the staphylococcal chromosomal cassette (SCCmec) [7]. Methicillin resistance in species such as S. epidermidis and *S. haemolyticus* is typically mediated by *mecA*, which encodes the modified penicillin-binding protein PBP2a, thereby allowing cell wall synthesis even in the presence of β-lactams [14,15]. These species also frequently exhibit multidrug resistance (MDR), highlighting their clinical relevance in orthopedic infections [19].

Efflux systems are protein-based transporters that contribute to resistance to antimicrobial agents, including antibiotics, disinfectants, and antiseptics [20]. The underlying mechanism involves the active extrusion of endogenous compounds, antimicrobial substances, heavy metals, and plant-derived molecules from the bacterial cell [21,22,23]. In addition to their broad substrate specificity, efflux pumps differ in the energy sources they use to drive transport [24]. Based on these characteristics, efflux systems are classified into six distinct families, of which only four are commonly associated with the genus *Staphylococcus* and linked to antibiotic resistance [25].

*S. epidermidis* has become one of the most clinically relevant species among CoNS. Several global studies report that approximately 80–90% of *S. epidermidis* strains isolated from patients with nosocomial infections carried the *mecA* gene [26,27]. The presence of *mecA* appears to be increased among biofilm-producing *S. epidermidis* isolates, thereby enabling these strains to exhibit enhanced resistance to multiple antibiotic classes [28].

During biofilm development, an initial monolayer of bacteria adheres to a biotic or abiotic surface. Attachment of staphylococci to extracellular host proteins is mediated primarily by fibronectin-binding proteins (fnbA, fnbB), fibrinogen-binding proteins (clfA, clfB), and proteins composed of serine–aspartate dipeptide repeats (SdrC, SdrD, SdrF), collectively known as MSCRAMMs (microbial surface component recognition adhesion matrix molecules) [29]. Virulence and biofilm formation ability are also affected by overexpression of the *srtA* gene, which encodes the transpeptidase sortase A and increases the rate at which these surface proteins are anchored to the cell wall during biosynthesis [30]. During biofilm aggregation and proliferation, extracellular matrix is produced, the main components of which is Polysaccharide Intercellular Adhesin (PIA), synthesized by genes of the *icaABCD* locus. Wall Teichoic Acids (WTAs) and extracellular DNA also contribute to biofilm aggregation [31]. Another gene whose products may be significantly involved in biofilm formation in staphylococci is *bap* (biofilm-associated protein). The *agrA* gene encodes a regulatory factor that is part of the agr (accessory gene regulator) quorum-sensing system. By regulating the expression of virulence factors and biofilm formation, it can significantly influence the bacterium’s ability to cause disease [32].

Biofilm formation further contributes to the persistence and chronicity of infections by protecting bacterial cells from host immune responses and antimicrobial therapy. Hospitalized patients, especially those in intensive care units or receiving prolonged antibiotic therapy, serve as a significant reservoir for these resistant, biofilm-forming strains [33].

The primary objective of our study is to synthesize current evidence on the prevalence of biofilm-forming, antibiotic-resistant CoNS isolated from biological materials in hospital settings, including blood cultures, catheters, urine, and swabs from wounds and skin lesions. In resistant isolates, the presence of efflux pumps, genes of methicillin resistance, and biofilm-associated genes was investigated.

## 2. Materials and Methods

### 2.1. Sample Collection, Cultivation, and Identification of CoNS

Over half a year, 68 patient samples from random screening conducted during hospitalization in the Orthopedics Department in Slovakia were analyzed. For screening during hospitalization, samples from skin swabs, wounds, blood, urine, catheter, and punctate were collected. From the collected samples, 28 MRCoNS strains were identified and used for further phenotypic and genotypic analyses.

All samples were processed and inoculated onto the following agar plates: blood agar (HiMedia Laboratories, Mumbai, India; cat. no. M073-500G), Mannitol Salt agar (HiMedia Laboratories, Mumbai, India, cat. no. M118-500G), and Baird-Parker agar (HiMedia Laboratories, Mumbai, India; cat. no. M073). Incubation was carried out at 37 °C for 24 h. After incubation, isolates exhibiting growth characteristics typical of the genus *Staphylococcus* were selected and subjected to Gram staining, which allowed their identification as Gram-positive cocci.

### 2.2. References of Strains

*S. aureus* CCM 4223 strain, isolated from a wound, was used as a reference for the detection of the 16S rRNA, *eap*, and *nuc* genes. Control reference strains for *mecA* and *mecC* detection were *S. aureus* CCM 4750 and *S. edaphicus* CCM 8731.

Control strains carrying genes associated with biofilm formation, including *agrA/srtA/icaD*, *icaA/icaB/icaC*, *bap*, *fnbA/fnbB*, and *clfA/clfB*, were employed. To evaluate the phenotypic ability to form biofilm, *Staphylococcus aureus* CCM 4223 was employed. At the same time, *Staphylococcus epidermidis* CCM 4418 was used as a non-biofilm-forming reference strain. All strains were obtained from the Czech Collection of Microorganisms (Brno, Czech Republic).

### 2.3. Genomic DNA Extraction

Bacterial DNA was extracted from an 18 h culture grown in modified Brain Heart Infusion (mBHI) broth (HiMedia Laboratories, Mumbai, India; cat. no. M210I) using the High Pure PCR DNA Extraction Kit (Roche Molecular Systems, Inc., Pleasanton, CA, USA; cat. no. 11796828001), following the manufacturer’s instructions. The purity and concentration of the extracted DNA were measured using a NanoDrop One spectrophotometer (Thermo Fisher Scientific, Madison, WI, USA; cat. no. 13-400-518) at 260 and 280 nm.

### 2.4. Genus and Species Identification of the Strains

The isolated strains were identified at the molecular level using confirmatory multiplex PCR (mPCR) to amplify the 16S rRNA gene segment of the *Staphylococcus* genus. Furthermore, this multiplex PCR (mPCR) was used to detect *S. aureus* by amplifying specific fragments of its *eap* (extracellular adhesion protein) and *nuc* (thermostable nuclease) genes. All staphylococcal isolates confirmed by mPCR were subsequently identified using MALDI-TOF mass spectrometry (version 2.0, BioTyper Library version 3.0; Bruker Daltonics, Billerica, MA, USA). The identified strains were preserved in Microbank cryovials (Pro-Lab, Mississauga, ON, Canada; cat. no. PL.170C/B) at −80 °C.

### 2.5. Antimicrobial Susceptibility Testing

The minimum inhibitory concentration (MIC) for assessing phenotypic antibiotic resistance was determined using the colorimetric microdilution method, as described by Gattringer et al. [34], with automatic reading facilitated by the Miditech system (Bel-Miditech s.r.o., Bratislava, Slovakia; cat. no. 002002). The antibiotics tested, in order, were ampicillin (AMP), ampicillin + sulbactam (SAM), oxacillin (OXA), cefoxitin (FOX), piperacillin + tazobactam (TZP), erythromycin (ERY), clindamycin (CLI), linezolid (LNZ), rifampicin (RIF), gentamicin (GEN), teicoplanin (TEC), vancomycin (VAN), trimethoprim (TMP), chloramphenicol (CHL), tigecycline (TGC), moxifloxacin (MFX), ciprofloxacin (CIP), tetracycline (TET), trimethoprim + sulfonamide (COT), and nitrofurantoin (NIT). MIC values for each antibiotic were interpreted according to the EUCAST (version 15.0) clinical breakpoints [35].

### 2.6. Biofilm Formation Testing

Biofilm activity was evaluated using a modified colorimetric method based on crystal violet staining, as described by O’Toole et al. [36]. A bacterial suspension was prepared from an overnight culture on blood agar and adjusted to a 1 McFarland standard. Subsequently, 100 µL of this suspension and 100 µL of mBHI were added to each well of a 96-well microtiter plate (Brand GMBH + CO KG, Wertheim, Germany; cat. no. 6233756). The plates were incubated for 24 h at 37 °C. After the incubation period, the medium was removed, and the wells were rinsed four times with distilled water. Biofilms were stained by adding 200 µL of 0.1% crystal violet solution (Merck, Darmstadt, Germany; cat. no. 101408) and incubated at room temperature for 30 min. Following the incubation period, each well received 200 µL of 30% glacial acetic acid. Optical density (OD) was measured at 550 nm using a Synergy 4 reader (Merck, Darmstadt, Germany). Averages were calculated based on three replicates of a single test strain. The reference strains included *S. aureus* CCM 4223 and *S. epidermidis* CCM 4418 (Czech Collection of Microorganisms, Brno, Czech Republic). Modified BHI was utilized as a negative control.

### 2.7. Detection of Genes Using PCR

The presence of genes responsible for antibiotic resistance was monitored by PCR using primers amplifying segments of the *mecA* and *mecC* genes. Biofilm-associated genes were monitored using singleplex PCR (*bap*) and mPCR (*icaABCD*, *srtA*, *agrA*, *fnbAB*, *clfAB*). The sequences of individual primers and the composition of the reaction mixture are visible in Table 1.

PCR and mPCR reactions were conducted using a T100™ Thermal Cycler (Bio-Rad Laboratories, Singapore; cat. no. 1861096). The PCR program included an initial denaturation step at 95 °C for 3 min, followed by 35 cycles of denaturation at 95 °C for 30 s, annealing at 61 °C for 30 s, and extension at 72 °C for 20 s. After the final cycle, a final extension step was performed at 72 °C for 10 min. Subsequently, PCR products were electrophoretically separated (Thermo Fisher Scientific, Marietta, OH, USA; Major Science, Saratoga, CA, USA) on a 2% agarose gel with the addition of GoodView^TM^ fluorescent dye (Amplia s.r.o., Bratislava, Slovakia), followed by visualization using a UV transilluminator (Major Science, Saratoga, CA, USA; cat. no. MUV21-254).

### 2.8. Detection of Efflux Pump

Efflux pump activity was evaluated on Mueller–Hinton agar (HiMedia Laboratories, Mumbai, India; cat. no. M173-500G) supplemented with 2.5 mg/L ethidium bromide in Petri plates, using the cartwheel method based on the extrusion of a fluorescent dye, as described by Martins et al. [44]. Each plate was divided into eight radial sectors (in a cartwheel pattern). A bacterial suspension adjusted to a 0.5 McFarland standard was prepared from an overnight culture in physiological saline. The strains were streaked from the center of the plate outward toward the periphery. Subsequently, the plates were incubated for 24 h at 37 °C and protected from light with aluminum foil. After incubation, the inoculated plates were examined using the UV-Reader Quantum system (UV light source; Vilber Lourmat, Collégien, France) and analyzed with the VisionCapt digital imaging system (Vilber Lourmat, Collégien, France). For quality control, *S. aureus* ATCC 25923 EtBr (efflux pump-producing strain) and *S. aureus* ATCC 25923 (non-efflux pump-producing strain), obtained from the American Type Culture Collection (Manassas, VA, USA), were used. Physiological saline alone served as the negative (purity) control.

### 2.9. Statistical Assessment

Biofilm formation was evaluated using statistical analysis performed in GraphPad Prism version 8.3.0 (GraphPad Software Inc., San Diego, CA, USA). A one-way ANOVA followed by Dunnett’s post hoc test was used to determine statistical significance, with *p* < 0.001 as the threshold.

Minimum inhibitory concentration data were additionally processed in the Miditech Analyser interpretation software version Expert 09/2024 (Bel-Miditech s.r.o., Bratislava, Slovakia; cat. no. 002002), which incorporates statistical analysis features.

## 3. Results

### 3.1. Identification of Isolates

Among the 28 isolates analyzed, all staphylococci were subsequently confirmed by mPCR. All isolates tested positive for a fragment of the 16S rRNA gene specific to the genus *Staphylococcus* (141 bp), which can be used for genus identification, thereby confirming them as staphylococci. Fragments of the *nuc* (103 bp) and *eap* (230 bp) genes, which are specific to *S. aureus* and serve to distinguish it from other staphylococci, were not detected in any of the examined isolates. These findings complemented and confirmed the results obtained from phenotypic analysis.

For accurate identification of staphylococci, the mass spectrometry method MALDI-TOF MS, was used. Based on the score values, these strains of coagulase-negative staphylococci (CoNS) were identified: *S. epidermidis* (*n* = 10), *S. hominis* (*n* = 8), *S. haemolyticus* (*n* = 4), *S. lugdunensis* (*n* = 3), while other species *S. simulans*, *S. pasteuri*, and *S. warneri* were detected only once.

### 3.2. Phenotypic Antimicrobial Resistance Profiles of CoNS

Minimal inhibitory concentrations (MICs) were determined for all CoNS isolates (*n* = 28) against 20 antibiotics (see Table 2). Among the 28 isolates, the highest resistance rates were observed to ampicillin, oxacillin, rifampicin, trimethoprim (100%), and erythromycin (62%).

The MICxG values for oxacillin were 9.7 mg/L, for moxifloxacin 0.3 mg/L, for erythromycin 4.0 mg/L, for clindamycin 0.67 mg/L, for rifampicin 0.49 mg/L, and for trimethoprim 32 mg/L, which exceeded the EUCAST clinical breakpoints of MIC [35].

**Figure 1:**
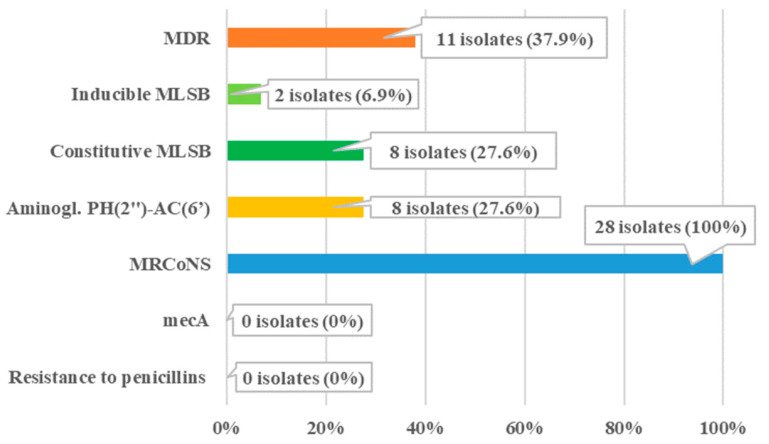
Phenotype resistance mechanisms in 28 isolates.

Almost all strains showed multiple resistance mechanisms simultaneously (Figure 1). The MRCoNS resistance mechanism was detected in 28 tested isolates. The phenotype mechanisms MDR was found in MRCoNS strains (11/28), mainly in *S. epidermidis*, *S. haemolyticus*, *S. hominis*, and *S. warneri*, mainly in hospitalized patients (9/11). The MLSB (macrolide–lincosamide–streptogramin B) resistance mechanism was additionally present in 10 MRCoNS isolates. The constitutive MLSB (MLSBc) resistance mechanism (8/28) was confirmed, while inducible MLSB (MLSBi) was detected in only two isolates. The most prevalent resistance mechanism was penicillin resistance, followed by aminoglycoside resistance of the PH(2″)-AC(6′) type (8/28). This type of resistance complicates treatment with these antimicrobial agents and is associated with combined enzymatic resistance to ampicillin, gentamicin, netilmicin, and tobramycin [45].

### 3.3. Analysis of Efflux Pump Expression

The capacity of individual clinical CoNS isolates to produce efflux pumps was evaluated using the ethidium bromide cartwheel technique described by Martins et al. [44]. As controls, *S. aureus* ATCC 25923EtBr served as the efflux-positive reference strain, whereas *S. aureus* ATCC 25923 was used as the efflux-negative reference strain [46].

**Figure 2:**
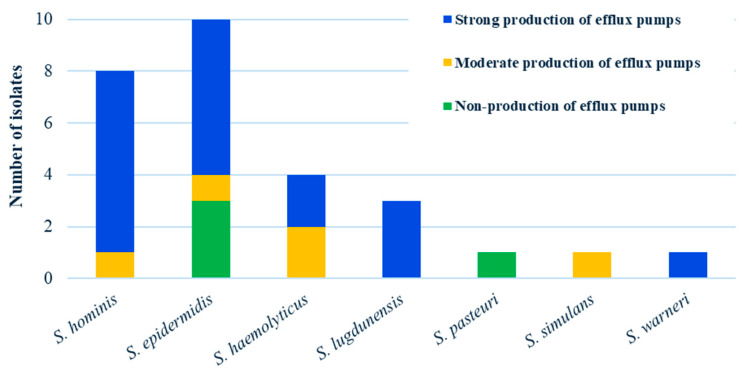
Classification of 28 clinical CoNS isolates by efflux pump production level (none, weak, intermediate, strong), presented by species.

Among the 28 clinical isolates analyzed, 14% showed no detectable efflux activity, comprising three *S. epidermidis* isolates and one *S. pasteuri* isolate. Intermediate efflux pump activity was observed in 18% of isolates. The predominant group consisted of strong efflux pump producers, accounting for 68% of all isolates (Figure 2).

**Figure 3:**
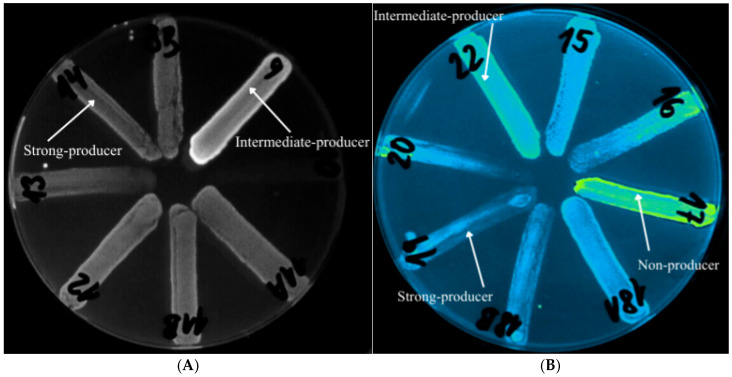
UV-based visualization of efflux pump activity on Mueller–Hinton agar containing ethidium bromide, presented as (**A**) grayscale and (**B**) color images.

Overall, the highest proportion of efflux pump-producing CoNS was found among *S. epidermidis*, *S. hominis,* and *S. haemolyticus* isolates (Figure 3A,B).

### 3.4. Detection of Antimicrobial Resistance Genes in CoNS Isolates

The *mecA* gene was detected in 12 analyzed isolates (from *n* = 28). *MecA* positivity was most frequently observed among MRCoNS strains, specifically in *S. hominis* (*n* = 6), *S. epidermidis* (*n* = 3), *S. haemolyticus* (*n* = 2), and *S. lugdunensis* (*n* = 1). Notably, half of the *mecA*-positive isolates (*n* = 6) exhibited an MDR phenotype (AMP, OXA, RIF, TMP), as shown in Table 1. As control strains, *S. aureus* CCM 4750 and *S. edaphicus* CCM 8731 were used as reference strains for *mecA* and *mecC* detection, respectively.

### 3.5. Assessment of Biofilm Formation

**Figure 4:**
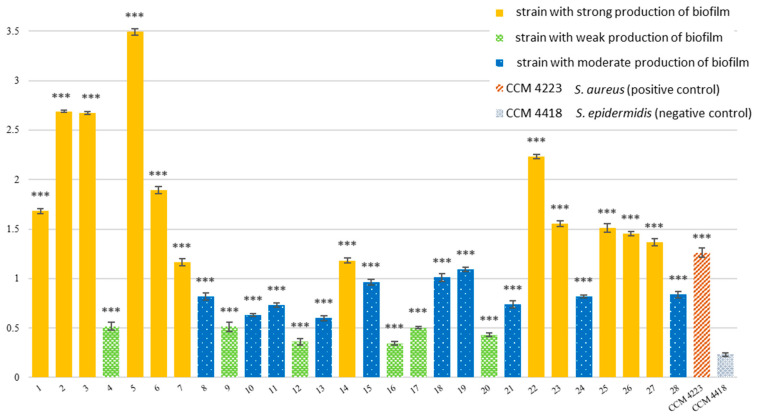
Biofilm formation in 28 clinical isolates. Description: Samples 1–28—strains with weak (green), moderate (blue), or strong (yellow) production of biofilm; *** significant production of biofilm *p* < 0.001; CCM 4223 *S. aureus*—positive control, biofilm-forming strain; CCM 4418 *S. epidermidis*—negative control, non-biofilm-forming strain.

Significant biofilm-forming ability was assessed in all 28 confirmed MRCoNS clinical isolates from hospitalized patients. Biofilm formation was evaluated using a colorimetric assay by O’Toole et al. [36], which revealed pronounced biofilm activity, as summarized in Figure 4. Statistically, it was confirmed that all investigated strains were significant biofilm producers (*p* < 0.001).

All isolates exhibited a pronounced capacity to form biofilm, comparable to the positive control—*S. aureus* reference strain (CCM 4223), and clearly distinct from the non-biofilm-forming reference strain *S. epidermidis* (CCM 4418).

Biofilm-associated genes were screened using singleplex PCR (*bap*) and multiplex PCR (*icaABCD*, *srtA*, *agrA*, *fnbAB*, *clfAB*). None of these biofilm-associated genes was detected in any of the examined isolates. Nevertheless, all 28 MRCoNS strains demonstrated strong, moderate, or weak biofilm-forming activity in phenotypic assays.

Notably, all strains demonstrated the phenotypic ability to form biofilms. Strong biofilm formation was observed in 12/28 MRCoNS isolates, moderate biofilm activity was detected in 10/28 isolates, week biofilm activity was detected in 6/28 isolates.

### 3.6. Correlation Between Antimicrobial Resistance, Resistance Genes, and Biofilm Formation

Interesting results were found in different samples, as illustrated and compared in Table A1. In three strains—two *S. haemolyticus* isolates (no. 3 and 26) and one *S. hominis isolate* (no. 23), both phenotypic and genotypic methicillin resistance (*mecA*) were concurrently confirmed. These strains, isolated from patients during hospitalization, also exhibited strong biofilm-forming capacity and phenotypic MDR, with two isolates additionally demonstrating resistance to aminoglycosides (PH(2″)-AC(6′) modification) and the MLSBc phenotype. In 12 isolates (no. 1, 2, 3, 7, 14, 16, 17, 20, 23, 25, 26, and 28) MDR, strong efflux pump activity, and strong or moderate biofilm formation were detected simultaneously.

In the remaining MRCoNS isolates (*n* = 3), namely *S. epidermidis* (no. 2), *S. hominis* (no. 14), and *S. haemolyticus* (no. 7), phenotypic and genotypic methicillin resistance (*mecA*) were also detected. Moreover, phenotypic resistance—MDR and MLSBc was confirmed in three of the strains of *S. epidermidis* (no. 2, 20) and *S. hominis* (no. 14). In addition, in strains *S. haemolyticus* (no. 7) and *S. hominis* (no. 14), aminoglycoside resistance of the PH(2″)-AC(6′) type was determined. In *S. haemolyticus* (no. 3), phenotypic MDR, MRCoNS, strong efflux pump activity, and strong biofilm production were observed, and the genotypic *mecA* gene was confirmed.

Notably, in two MRCoNS strains, namely *S. simulans* (no. 10) and *S. hominis* (no. 28), the phenotypic resistance mechanism MLSBi was confirmed. In addition, *S. hominis* (no. 28) isolated during hospitalization, exhibited MDR, including aminoglycoside resistance. Both of these strains demonstrated moderate biofilm-forming activity.

In five MRCoNS strains exhibiting both phenotypic and genotypic presence of *mecA,* no additional resistance mechanisms were detected. Among these strains isolated during hospitalization, *S. lugdunensis* (no. 4) displayed weak biofilm-forming activity, whereas *S. epidermidis* (no. 6) exhibited strong biofilm-forming activity.

## 4. Discussion

CoNS have emerged in recent years as clinically significant opportunistic pathogens. These organisms are frequently implicated in various healthcare-associated infections, with bloodstream infections and prosthetic device-related infections being the most common and clinically consequential manifestations [33].

Our study provides a comprehensive overview of CoNS colonization and infection dynamics throughout the perioperative period in an orthopedic clinic at a Slovak hospital over a half-year period.

CoNS continue to rise in prominence as key pathogens in prosthetic joint infections and implant-associated orthopedic infections. Epidemiological studies consistently identify *S. epidermidis* as the most frequently isolated species, followed by *S. haemolyticus*, *S. lugdunensis*, *S. capitis*, and other less common species such as *S. warneri* or *S. hominis* [47].

*S. epidermidis*, *S. lugdunensis*, *S. saprophyticus*, and *S. schleiferi* are the most frequently identified MRCoNS in orthopedic infections. In contrast, some less common species, such as *S. capitis*, may be absent depending on the study population and local epidemiology. CoNS are key etiological agents of multiple severe infections, including osteomyelitis, otitis, wound infections, endophthalmitis, urinary tract infections, meningitis, and pneumonia [13,48]. In a large periprosthetic joint infection study (*n* = 215 CoNS strains), *S. epidermidis* accounted for 60% of isolates, with *S. capitis*, *S. lugdunensis*, *S. warneri*, *S. hominis*, and *S. haemolyticus* making up the remainder. These findings highlight the predominance of *S. epidermidis* in device-associated infections and emphasize the diversity of CoNS species contributing to periprosthetic joint infections [49]. Our results from clinical samples show a very similar representation of CoNS strains (*S. haemolyticus*, *S. epidermidis*, and *S. lugdunensis*). In comparison with our study, among the 28 MRCoNS strains identified, *S. epidermidis* was the most prevalent species (*n* = 10), followed by *S. hominis* (*n* = 8), *S. haemolyticus* (*n* = 4), *S. lugdunensis* (*n* = 3), *S. warneri* (*n* = 1), *S. pasteuri* (*n* = 1), and *S. simulans* (*n* = 1). Notably, *S. capitis* was not detected. Although our study included only 28 CoNS isolates, 12 were MDR, a proportion comparable to that reported in larger studies of 215 isolates [49]. Similarly, strong or moderate biofilm formation was observed in 22 of our isolates, consistent with observations in larger populations, highlighting the clinical significance of these phenotypes even in a smaller sample set.

Modak et al. [50] demonstrated that *S. haemolyticus* (43.2%), *S. epidermidis* (27.2%), *and S. hominis* (14.8%) were the most frequently isolated MRCoNS species, with widespread MDR. Most isolates in that study retained susceptibility to linezolid (84%) but were completely resistant to penicillin.

Overuse of antibiotics, in association with a history of multiple hospitalization and exposure to medical devices, plays a pivotal role as a risk factor for the development of antimicrobial resistance [33]. In the study by Elifanii et al. [51], 168 samples from hospitalized patients in an orthopedic department were examined, with 40 isolates identified as CoNS. The antibiotics with the greatest sensitivity to CoNS were vancomycin, linezolid, tigecycline, moxifloxacin, and nitrofurantoin. However, the highest resistance was recorded to oxacillin (87.5%), ampicillin (85.7%), erythromycin (55%), ciprofloxacin (45.5%), and levofloxacin (45%).

According to ECDC data and European surveillance reports, the most frequently reported form of antibiotic resistance among CoNS in Europe is methicillin/oxacillin resistance (MRCoNS), which is most commonly observed in clinical isolates [52]. The most prevalent phenotype mechanism of resistance in *S. hominis*, *S. haemolyticus*, and *S. epidermidis* isolates was the constitutive MLSB resistance type [53].

In parallel, in our study, MRCoNS isolates exhibited MDR (in 11 strains), with the highest resistance rates observed against ampicillin, oxacillin, rifampicin, trimethoprim (100%), and erythromycin (62%). The MLSB resistance mechanism was present in 10 MRCoNS isolates. These results underscore the persistent challenge of antimicrobial resistance in CoNS, particularly among hospitalized patients and those receiving long-term antibiotic therapy.

Kitota [54] investigated four staphylococcal species (*S. epidermidis*, *S. haemolyticus*, *S. saprophyticus*, and *S. hominis*) from blood, tissue, and swabs. Of the 40 isolates, MRCoNS were recoverable only in *S. haemolyticus* and *S. hominis*. The *mecA* gene was detected in thirty-eight isolates (95%), whereas the remaining two (5%) lacked both *mecA* and *mecC*. In our study, the *mecA* gene was detected in 12 analyzed isolates (from *n* = 28). In the remaining MRCoNS isolates, neither *mecA* nor *mecC* was detected. *MecA* positivity was most frequently observed among MRCoNS strains, specifically in *S. hominis* (*n* = 6), *S. epidermidis* (*n* = 3), *S. haemolyticus* (*n* = 2), *and S. lugdunensis* (*n* = 1). In contrast, isolates from our study showed lower *mecA* prevalence in *S. hominis* (38%), *S. haemolyticus* (22%), and *S. epidermidis* (7%).

The activity of efflux pumps in CoNS has not yet been extensively investigated. The available literature predominantly focuses on molecular-level identification, with studies targeting efflux systems belonging to the MFS family, the RND family, and the SMR family [55,56,57].

Klempt et al. [58] reported the presence of efflux-associated *tet* genes in 79% of CoNS isolates, whereas Srirama et al. [20] detected *qacA/B* genes in 27.02% and *smr* genes in 4.1% of CoNS isolates.

However, these findings are not directly comparable with ours, as they represent genotypic detection of specific efflux-related genes, rather than general phenotypic assessment, which does not differentiate between efflux pump families. Nevertheless, for preliminary rapid diagnostic purposes, phenotypic approaches appear to be more practical and efficient. To date, *S. aureus*—a coagulase-positive species—remains the most extensively studied staphylococcal organism with regard to efflux-mediated resistance [14,57,59]. The ethidium bromide cartwheel assay has been applied phenotypically to staphylococci, who reported efflux activity in 83% of *Staphylococcus* spp. isolates, although no species-specific characterization was provided [57]. These findings are consistent with our results, as we identified efflux pump production in 84% of isolates (68% strong and 18% intermediate producers), with *S. epidermidis* and *S. hominis* representing the most prevalent efflux-producing species. To the best of our knowledge, additional phenotypic data on efflux mechanisms in CoNS have not yet been published.

Although MRCoNS isolates in our study exhibited phenotypically varying degrees of biofilm formation, none carried biofilm-associated genes. This finding suggests that biofilm development in these strains may be driven by alternative, gene-independent mechanisms, highlighting the complexity of biofilm-mediated resistance and its potential clinical implications.

Thirteen isolates (46.7%) showed the presence of one gene, six (20%) isolates exhibited the two genes, while ten (33.3%) had neither of them. The formation of staphylococci biofilms in the absence of *ica* genes may be related to the presence of other ica-independent biofilm formation mechanisms [60]. In our former study [61], where we studied *S. aureus* from skin lesions in human patients, we found high resistance to ampicillin, gentamicin, erythromycin, and clindamycin. All studied isolates (*n* = 13) showed the ability to form biofilms (presence of *icaABCD*, *agrA*, *srtA*, *clfAB*, and *fnbAB* genes).

Although all 28 CoNS isolates in our study were phenotypically strong, moderate, and weak biofilm producers, classical biofilm-associated genes (*icaABCD*, *agrA*, *srtA*, *fnbAB*, *clfAB*, *bap)* were not detected. This discrepancy is likely due to alternative biofilm mechanisms in CoNS, including PIA-independent adhesins such as *Aap* and *Embp*, MSCRAMMs, and other cell surface factors that mediate intercellular adhesion and biofilm accumulation [62]. Comparative genomic studies further indicate that biofilm formation has evolved redundantly and independently across different *Staphylococcus* species; therefore, the absence of canonical genes does not preclude a strong biofilm phenotype [63,64].

Interestingly, all CoNS isolates examined for biofilm production lacked classical biofilm-associated genes (*icaABCD*, *bap*, *agrA*, *srtA*, *fnbAB*, *clfAB*), yet formed robust biofilms. Biofilm genes are often found on mobile elements. In the hospital environment, they may be lost or silenced if biofilm formation occurs by alternative mechanisms, including extracellular DNA, regulators, modification of surface proteins, production of extracellular polysaccharides or proteins through alternative pathways, and adaptive stress responses. Environmental factors such as subinhibitory antibiotics, disinfection, surface properties, or host immune pressure can trigger these pathways, leading to robust biofilm formation without biofilm-associated genes [65], highlighting the phenotypic plasticity of CoNS and suggesting that genotypic screening alone may underestimate their virulence. Additionally, environmental pressures typical of hospital and implant-associated settings can trigger biofilm development independently of these genes [66]. These studies confirm that the absence of biofilm genes does not mean the absence of biofilm.

In our study, MDR, strong efflux pump activity, and strong or moderate biofilm formation were simultaneously observed in 12 MRCoNS isolates. The co-occurrence of these traits in a substantial proportion of the isolates highlights the adaptive potential of CoNS and underscores the importance of evaluating multiple resistance and virulence mechanisms in parallel rather than in isolation [9]. Previous studies have typically investigated MDR, efflux pump activity, or biofilm formation as separate phenomena; however, their combined presence may substantially enhance bacterial persistence and contribute to treatment failure. This effect is partly explained by the role of efflux pumps in reducing antimicrobial susceptibility and their association with robust biofilm formation, which further limits antibiotic efficacy in pathogenic staphylococci and other bacteria. Notably, the majority of CoNS isolates carried the *mecA* gene, which is associated with methicillin resistance and increased persistence in biofilm-associated staphylococcal infections [67,68].

In the study by Seng et al. [69], biofilm genes (*bap*, *icaABCD*) were not detected in MRCoNS isolates from the hospital environment, despite their biofilm production and *mecA* gene carriage.

Because biofilm formation and antimicrobial resistance frequently co-occur, often as a consequence of shared regulatory mechanisms, horizontal gene transfer, and increased stress tolerance, their surveillance is essential for accurate epidemiological assessment and effective infection control [70].

## 5. Conclusions

The antimicrobial resistance observed in CoNS in orthopedic clinics represents a critical challenge for modern medicine and significantly impacts current and future therapeutic strategies.

In conclusion, MRCoNS strains in hospitalized patients carrying the *mecA* gene exhibited a strong biofilm-forming phenotype despite lacking commonly investigated biofilm-associated genes *icaABCD*, *fnbAB*, *clfAB*, and *bap*. As our results show, vancomycin is still a reasonable choice for the treatment of severe infections due to MDR staphylococci.

There is a need for surveillance of nosocomial isolates of CoNS for resistance to beta-lactams, glycopeptides, and macrolides.

Our findings highlight the problems with biofilm-forming, resistant CoNS in hospitalized patients and the importance of diagnostics, separation, and quick treatment of patients, and keeping proper hygiene in hospitals (surfaces and air) to limit the spread of resistance to other departments.

## Figures and Tables

**Table 1:** List of primers and amplicons used for PCR analysis.

Gene	Primer Sequence (5′ → 3′)	Product Size (bp)	Reference
16S rRNA	TACAATGGACAATACAAAGGGC TCACCGTAGCATGCTGATCT	141	[37]
*eap*	TACTAACGAAGCATCTGCC TTAAATCGATATCACTAATACCTC	230	[38]
*nuc*	ACCTGCGACATTAATTAAAGCG TGTTTCAGGTGTATCAACCAATAATAG	103	[39]
*mecA*	TGGAAGTTAGATTGGGATCATAGC CGATGCCTATCTCATATGCTGTT	154	[39]
*mecC*	GACGATGGATCTGGTACAGCA CATTCATGAATGGATAAACATCGTA	94	[39]
*bap*	TTGACGAGGTTGGTAATGGC CGCCTACAGTTTCTGGTAATGC	87	[39]
*icaA*	CTTGCTGGCGCAGTCAATAC GTAGCCAACGTCGACAACTG	75	[40]
*icaB*	ATACCGGCGACTGGGTTTAT ATGCAAATCGTGGGTATGTGT	141	[41]
*icaC*	CTTGGGTATTTGCACGCATT GCAATATCATGCCGACACCT	209	[42]
*icaD*	ACCCAACGCTAAAATCATCG GCGAAAATGCCCATAGTTTC	211	[41]
*srtA*	GTGGTACTTATCCTAGTGGCAGC GCCTGCCACTTTCGATTTATC	183	[43]
*agrA*	TCGTAAGCATGACCCAGTTG AAATCCATCGCTGCAACTTT	96	[43]
*fnbA*	GAAGTGGCACAGCCAAGAAC ACGTTGACCAGCATGTGG	192	[43]
*fnbB*	CAATGATCCTATCATTGAGAAGAGTG CCTTCTACACCTTCAACAGCTGTA	156	[43]
*clfA*	GAGAGCATTTAGTTTAGCGGCA TCACCTTTAACAGCAGAATTAGGC	180	[39]
*clfB*	GTCTACACAAACGAGCAATACCAC TGAGGAACAGTTTGATCTTGCA	120	[39]

**Table 2:** Antimicrobial susceptibility profile of CoNS isolates. Resistance to different antimicrobials is highlighted.

	Identification	AMP	SAM	TZP	OXA	FOX	GEN	CIP	MFX	ERY	CLI	LNZ	RIF	VAN	TEC	TET	TGC	CHL	TMP	COT	NIT
1	*S. hominis*	4	2	4	8	32	0.5	0.5	0.125	1	0.25	2	1	1	0.125	0.25	0.063	2	32	2	4
2	*S. epidermidis*	8	2	4	8	32	0.5	0.25	0.125	16	8	1	0.5	4	2	2	0.25	8	32	4	8
3	*S. haemolyticus*	64	32	128	8	32	16	8	2	16	8	1	8	2	0.5	1	0.25	4	32	8	8
4	*S. lugdunensis*	16	2	2	8	32	0.5	0.25	0.25	0.5	0.25	1	0.5	1	0.125	0.25	0.031	1	32	4	8
5	*S. pasteuri*	4	2	1	8	16	0.5	0.125	0.063	1	0.25	2	0.5	0.5	0.125	0.5	0.063	2	32	2	8
6	*S. epidermidis*	8	2	1	8	32	1	0.125	0.125	8	0.25	1	0.125	1	2	1	0.125	2	32	4	8
7	*S. haemolyticus*	64	32	128	8	32	16	8	8	16	0.25	2	0.25	2	1	0.5	0.125	4	32	8	8
8	*S. epidermidis*	4	4	2	8	32	0.5	0.125	0.063	8	0.25	1	0.125	1	0.5	0.25	0.063	2	32	4	1
9	*S. hominis*	8	2	4	8	32	0.5	0.25	0.25	16	0.25	1	1	1	0.25	0.25	0.063	2	32	1	8
10	*S. simulans*	4	2	2	8	32	0.5	0.125	0.063	8	1	2	0.25	0.5	0.25	0.25	0.063	4	32	2	8
11	*S. epidermidis*	8	2	0.5	8	32	0.5	0.125	0.125	0.25	0.25	1	1	0.5	0.125	0.25	0.063	1	32	2	4
12	*S. epidermidis*	4	2	2	8	32	0.5	1	0.25	8	0.25	1	0.25	0.5	0.125	0.5	0.125	1	32	8	8
13	*S. hominis*	8	2	0.5	8	32	0.5	0.125	0.125	1	0.25	2	0.25	0.5	0.125	0.5	0.125	4	32	1	8
14	*S. hominis*	32	8	32	8	32	8	8	8	16	8	1	8	1	0.5	32	0.125	2	32	8	4
15	*S. hominis*	4	2	2	8	32	0.5	0.25	0.063	0.5	0.25	2	0.25	0.5	0.25	0.25	0.063	4	32	8	8
16	*S. warneri*	8	2	1	8	32	8	0.5	0.5	16	2	1	4	1	4	1	0.063	2	32	8	32
17	*S. hominis*	4	2	2	8	32	16	0.25	0.063	8	0.25	1	0.125	1	0.125	0.25	0.063	2	32	8	8
18	*S. epidermidis*	8	2	4	8	32	0.5	0.125	0.125	0.5	0.25	1	0.25	0.5	0.125	0.25	0.063	2	32	1	4
19	*S. epidermidis*	8	4	2	8	32	0.5	0.5	0.125	1	0.25	1	1	1	0.25	0.25	0.063	2	32	4	4
20	*S. epidermidis*	8	2	2	8	32	16	8	4	16	8	1	0.25	1	0.5	1	0.125	64	32	8	16
21	*S. lugdunensis*	4	2	2	8	32	0.5	0.25	0.25	0.5	0.25	1	0.5	1	0.5	0.125	0.063	2	32	1	4
22	*S. epidermidis*	16	2	1	8	32	0.5	0.25	0.063	1	0.125	1	0.25	1	2	0.25	0.063	2	32	4	8
23	*S. haemolyticus*	16	2	4	8	32	2	8	2	16	8	1	0.25	1	1	32	2	4	32	8	8
24	*S. epidermidis*	4	4	1	8	32	0.5	0.125	0.063	8	4	1	0.25	2	0.5	0.25	0.125	4	32	1	8
25	*S. haemolyticus*	8	4	1	8	32	1	0.25	0.063	16	0.25	1	0.125	1	2	32	0.125	4	32	1	8
26	*S. hominis*	64	32	128	8	32	8	8	8	16	8	1	8	1	2	1	0.25	4	32	8	8
27	*S. lugdunensis*	16	4	4	8	32	0.5	0.25	0.125	0.5	0.25	1	0.25	1	0.25	0.25	0.031	2	32	2	4
28	*S. hominis*	32	16	128	8	32	4	4	8	16	1	1	0.25	1	0.25	0.5	0.125	4	32	8	8

Description: AMP = ampicillin, SAM = ampicillin + sulbactam, TZP = piperacillin + tazobactam, OXA = oxacillin, FOX = cefoxitin, GE*n* = gentamicin, CIP = ciprofloxacin, MFX = moxifloxacin, ERY = erythromycin, CLI = clindamycin, LNZ = linezolid, RIF = rifampicin, VA*n* = vancomycin, TEC = teicoplanin, TET = tetracycline, TGC = tigecycline, CHL = chloramphenicol, TMP = trimethoprim, COT = trimethoprim + sulphonamide, and NIT = nitrofurantoin. Values highlighted in green represent resistance to the given antimicrobial agents.

## Data Availability

The original contributions presented in this study are included in the article. Further inquiries can be directed to the corresponding author.

## Abbreviations

The following abbreviations are used in this manuscript:

*agrA*	accessory gene regulator
*PH(2″)-AC(6′) type*	aminoglycoside resistance
*icaABCD*	the coding gene for polysaccharide intercellular adhesion (PIA)
*FnBPA*	fibronectin-binding protein A
*FnBPB*	fibronectin-binding protein B
*bap*	biofilm-associated protein
AMP	ampicillin
CIP	ciprofloxacin
CLI	clindamycin
CoNS	coagulase-negative staphylococci
COT	trimethoprim + sulphonamide
DNA	deoxyribonucleic acid
*clfA*	clumping factor A
*clfB*	clumping factor B
ERY	erythromycin
EUCAST	European Committee on Antimicrobial Susceptibility Testing
FOX	cefoxitin
GEN	gentamicin
CHL	chloramphenicol
LNZ	linezolid
MALDI-TOF MS	matrix-assisted laser desorption ionization–time of flight mass spectrometry
mBHI	modified brain heart infusion broth
MFX	moxifloxacin
MIC	minimum inhibitory concentration
MLSB_c_	constitutive resistance to macrolidex–lincosamide–streptogramin B
MLSB_i_	inducible resistance to macrolide–lincosamide–streptogramin B
MRCoNS	methicillin-resistant coagulase-negative staphylococci
MSCRAMM	microbial surface components recognizing adhesive matrix molecules
MDR	multidrug resistance
NIT	nitrofurantoin
OXA	oxacillin
PCR	polymerase chain reaction
PIA	polysaccharide intercellular antigen
RIF	rifampicin
SAM	ampicillin + sulbactam
SCC*mec*	staphylococcal cassette chromosome *mec*
*srtA*	gene for encoding sortase A
TEC	teicoplanin
TET	tetracycline
TGC	tigecycline
TMP	trimethoprim
TZP	piperacillin + tazobactam
VAN	vancomycin

## Appendix A

**Table A1:** Comparison results from phenotypic and genotypic analysis in 28 MRCoNS isolates.

Sample		Specimen	Biofilm Formation	Efflux Pumps	Phenotypic Analysis				Genotypic Analysis														
No.	Identification				MRCoNS	MLSB	AMI	MDR	16S rRNA	*eap*	*nuc*	*mecA*	*mecC*	*icaA*	*icaB*	*icaC*	*icaD*	*agrA*	*srtA*	*fnbA*	*fnbB*	*clfA*	*clfB*
1	*S. hominis*	skin	strong	strong	+	−	−	−	+	−	−	−	−	−	−	−	−	−	−	−	−	−	−
2	*S. epidermidis*	blood	strong	strong	+	+_c_	−	+	+	−	−	−	−	−	−	−	−	−	−	−	−	−	−
3	*S. haemolyticus*	catheter	strong	strong	+	+_c_	+	+	+	−	−	+	−	−	−	−	−	−	−	−	−	−	−
4	*S. lugdunensis*	wound	weak	strong	+	−	−	−	+	−	−	+	−	−	−	−	−	−	−	−	−	−	−
5	*S. pasteuri*	blood	strong	negative	+	−	−	−	+	−	−	−	−	−	−	−	−	−	−	−	−	−	−
6	*S. epidermidis*	wound	strong	negative	+	−	−	−	+	−	−	+	−	−	−	−	−	−	−	−	−	−	−
7	*S. haemolyticus*	skin	strong	strong	+	−	+	+	+	−	−	−	−	−	−	−	−	−	−	−	−	−	−
8	*S. epidermidis*	skin	moderate	strong	+	−	−	−	+	−	−	+	−	−	−	−	−	−	−	−	−	−	−
9	*S. hominis*	skin	weak	strong	+	−	−	−	+	−	−	+	−	−	−	−	−	−	−	−	−	−	−
10	*S. simulans*	skin	moderate	moderate	+	+_i_	−	−	+	−	−	−	−	−	−	−	−	−	−	−	−	−	−
11	*S. epidermidis*	skin	moderate	strong	+	−	−	−	+	−	−	−	−	−	−	−	−	−	−	−	−	−	−
12	*S. epidermidis*	skin	weak	strong	+	−	−	−	+	−	−	−	−	−	−	−	−	−	−	−	−	−	−
13	*S. hominis*	skin	moderate	strong	+	−	−	−	+	−	−	+	−	−	−	−	−	−	−	−	−	−	−
14	*S. hominis*	blood	strong	strong	+	+_c_	+	+	+	−	−	−	−	−	−	−	−	−	−	−	−	−	−
15	*S. hominis*	skin	moderate	strong	+	−	−	−	+	−	−	+	−	−	−	−	−	−	−	−	−	−	−
16	*S. warneri*	wound	weak	strong	+	+_c_	+	+	+	−	−	−	−	−	−	−	−	−	−	−	−	−	−
17	*S. hominis*	wound	weak	strong	+	−	+	+	+	−	−	+	−	−	−	−	−	−	−	−	−	−	−
18	*S. epidermidis*	skin	moderate	moderate	+	−	−	−	+	−	−	−	−	−	−	−	−	−	−	−	−	−	−
19	*S. epidermidis*	skin	moderate	negative	+	−	−	−	+	−	−	−	−	−	−	−	−	−	−	−	−	−	−
20	*S. epidermidis*	skin	weak	strong	+	+_c_	+	+	+	−	−	+	−	−	−	−	−	−	−	−	−	−	−
21	*S. lugdunensis*	wound	moderate	strong	+	−	−	−	+	−	−	−	−	−	−	−	−	−	−	−	−	−	−
22	*S. epidermidis*	skin	strong	strong	+	−	−	−	+	−	−	−	−	−	−	−	−	−	−	−	−	−	−
23	*S. haemolyticus*	urine	strong	moderate	+	+_c_	−	+	+	−	−	+	−	−	−	−	−	−	−	−	−	−	−
24	*S. epidermidis*	skin	moderate	negative	+	+_c_	−	−	+	−	−	−	−	−	−	−	−	−	−	−	−	−	−
25	*S. haemolyticus*	wound	strong	moderate	+	−	−	+	+	−	−	−	−	−	−	−	−	−	−	−	−	−	−
26	*S. hominis*	urine	strong	moderate	+	+_c_	+	+	+	−	−	+	−	−	−	−	−	−	−	−	−	−	−
27	*S. lugdunensis*	punctate	strong	strong	+	−	−	−	+	−	−	−	−	−	−	−	−	−	−	−	−	−	−
28	*S. hominis*	blood	moderate	strong	+	+_i_	+	+	+	−	−	+	−	−	−	−	−	−	−	−	−	−	−

Description: MRCoNS—methicillin−resistant coagulase-negative staphylococci; AMI—aminoglycoside resistance, PH(2″)-AC(6′) type; MDR—multidrug resistance; +_c_—MLSB_C_ (constitutive resistance to macrolide–lincosamide–streptogramin B); +_i_—MLSB_i_ (inducible resistance to macrolide–lincosamide–streptogramin B).
